# Producing Coral Offspring with Cryopreserved Sperm: A Tool for Coral Reef Restoration

**DOI:** 10.1038/s41598-017-14644-x

**Published:** 2017-10-31

**Authors:** Mary Hagedorn, Virginia L. Carter, E. Michael Henley, Madeleine J. H. van Oppen, Rebecca Hobbs, Rebecca E. Spindler

**Affiliations:** 1 Center for Species Survival, Smithsonian Conservation Biology Institute, Smithsonian Institution, Front Royal, VA 22630 USA; 2 Hawaii Institute of Marine Biology, University of Hawaii, Kaneohe, HI 96744 USA; 3 Australian Institute of Marine Science, Cape Cleveland, 4810 QLD Australia; 4 School of Biosciences, The University of Melbourne, Parkville, Melbourne, 3010 Victoria, QLD Australia; 5 Taronga Conservation Society Australia, Mosman, NSW 2088 Australia

## Abstract

Cryopreservation is an important conservation tool, which may help reef-building coral survive. However, scaling-up from small, laboratory-sized experiments to higher-throughput restoration is a major challenge. To be an effective restoration tool, the cryopreservation methods and husbandry to produce new offspring must be defined. This study examined small and larger-scale *in vitro* reproduction and settlement for *Acropora tenuis* and *Acropora millepora* and found that: 1) cryopreservation of coral sperm reduced sperm motility and fertilization success in half, thus fresh sperm, capable of becoming highly motile, is key; 2) the sperm-to-egg ratio and the concentration of the cryoprotectant treatments affected fertilization success in small- and larger-scale reproduction trials using cryopreserved sperm (p < 0.05); 3) cryopreservation did not affect settlement success, as larvae produced with fresh or cryopreserved sperm had the same settlement success (p > 0.05); and 4) the residence time of the sperm within the bank was not important as the fertilization success of sperm frozen for less than 1 month was similar to that frozen up to 2 years (p > 0.05). These results described the first settlement for coral larvae produced from cryopreserved sperm and established important ground-work principles for the use of cryopreserved coral sperm for future reef restoration efforts.

## Introduction

The overuse of fossil fuels is producing CO_2_, creating both a warmer and more acidic oceans, leading to coral stress and the greater likelihood of disease and bleaching^1–3^. Corals tolerate only a slight shift in the upper limit of physiological temperature tolerance and different species have differing tolerances to ocean warming. Acidification may slow coral growth^4^, and both bleaching and disease may lead to widespread loss of coral. However, bleaching, and its often related sweeping disease and stress-related events, may present the most immediate short-term concern, because of its global reach, its increasingly intensifying outbreaks and its extremely detrimental effect on reproduction. These observed and predicted losses for coral populations from both global and local warming events, and their concomitant losses in reproduction, erode population numbers, resulting in a potentially staggering loss in species diversity.

One way by which coral biodiversity can be preserved is cryopreservation; it shows great promise for securing the survival and genetic diversity of coral reefs for centuries, in a practical, cost-effective fashion. A number of coral holobiont cell types have been successfully cryopreserved, such as *Symbiodinium* from certain clades^5^ and coral sperm^6^, and these frozen samples have been stored in biorepositories around the world, such as the Taronga Zoo’s CryoDiversity Bank^7^. Additionally, these frozen samples have been used to fertilize fresh coral eggs and create coral larvae^6^. The power of coral biorepositories lies in their proven potential for protecting and maintaining biodiversity, which can be used to potentially prevent extinctions and reseed coral reefs worldwide. Additionally, the cells in these biobanks, frozen but alive, are treasure-troves of knowledge about reef DNA. Today, there are a variety of new ideas to help corals become more resilient, such as assisted evolution^8,9^, assisted gene flow^10,11^ and assisted colonisation^12,13^. Once effective, many of these newer restoration tools will require the use of cryopreserved samples, especially if hybridization is desired between species with very different spawning times.

When considering cryopreservation needs for coral restoration around the world, one of the major challenges we face is scaling up for high-throughput reproduction from smaller, laboratory-scale experiments. For a biorepository to be part of a conservation management tool, the use of the cryopreserved material and the husbandry to produce new offspring must be well defined. We have developed methods to collect coral adults, maintain them in captivity, fertilize eggs with fresh and thawed sperm and rear their offspring successfully; however, a comparison of the settlement success for larvae produced with fresh and cryopreserved sperm is lacking. In most of our previous work, we used small experimental *in vitro* cultures (5 ml, consisting of ~50 eggs), but, given the high mortality associated with the early life stages of coral reproduction, effective conservation requires the production 10 s or 100 s of thousands of settled larvae. In this paper we define the *in vitro* methods for two species of reef-building acroporid coral, *Acropora tenuis* and *A. millepora*. Specifically, we examined: 1) small-scale *in vitro* culture and sperm concentrations for using cryopreserved sperm effectively for these species; 2) fertilization success with varying sperm-to-egg ratios for fresh and cryopreserved sperm in the *in vitro* culture system for small- and larger-scale fertilization processes; and, 3) the effective settlement success of coral produced with fresh and cryopreserved sperm that had been in liquid nitrogen storage for different periods of time.

## Results

### Experiment 1: Sperm-to-Egg Ratio and Cryoprotectant Concentration Affect Small and Larger-Scale *In vitro* Fertilization Success

**Figure 1:**
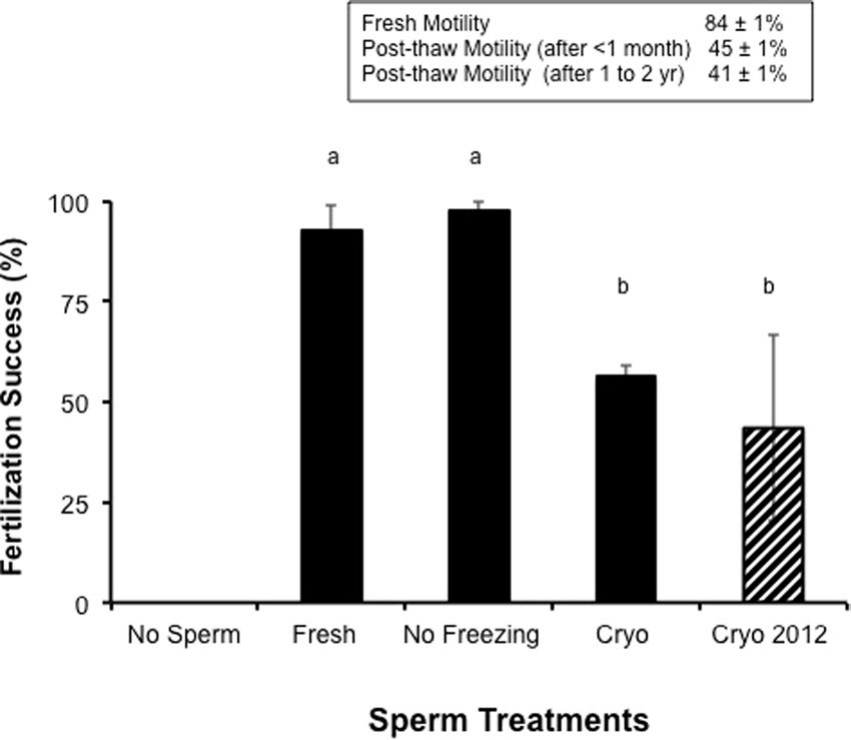
Mean fertilization success of *A. tenuis* (n = 2 egg donors and 4 to 6 pooled sperm donors, 22 to 75 eggs/treatment) and *A. millepora* (n = 2 egg donors and n = 4 to 6 pooled sperm donors, 14 to 114 eggs/treatment), using a 5 ml *in vitro* system. The fertilization success for both species was pooled and the relative success of the five treatments examined. The five treatments included no sperm added, fresh sperm, no freezing (sperm just exposed to 10% DMSO) and sperm exposed to 10% dimethyl sulfoxide and cryopreserved for less than 1 month and over 1 to 2 yr (striped bar). Bars represent means (±SE) and different letters indicated differences in the mean (p < 0.05, ANOVA, F = 17.34).

Small scale *in vitro* experiments revealed a great deal about reproduction. The fertilization success using fresh and cryopreserved sperm from *A. tenuis* and *A. millepora* was different (p < 0.05, ANOVA, F = 17.3). The fresh sperm for the two species demonstrated a > 90% fertilization success, whereas cryopreservation reduced the fertilization success in half (Fig. 1). Exposing the sperm to the cryoprotectant alone did not reduce the fertilization success (p > 0.05, ANOVA, F = 17.3), but combining the cryoprotectant exposure with freezing did (p < 0.05, Fig. 1). Finally, the frozen sperm banked for years had the same fertilization success as that banked for days to months (p > 0.05, ANOVA, F = 17.3). Thus, cryopreservation impacted the motility, reducing the fertilization success of these coral.

**Figure 2:**
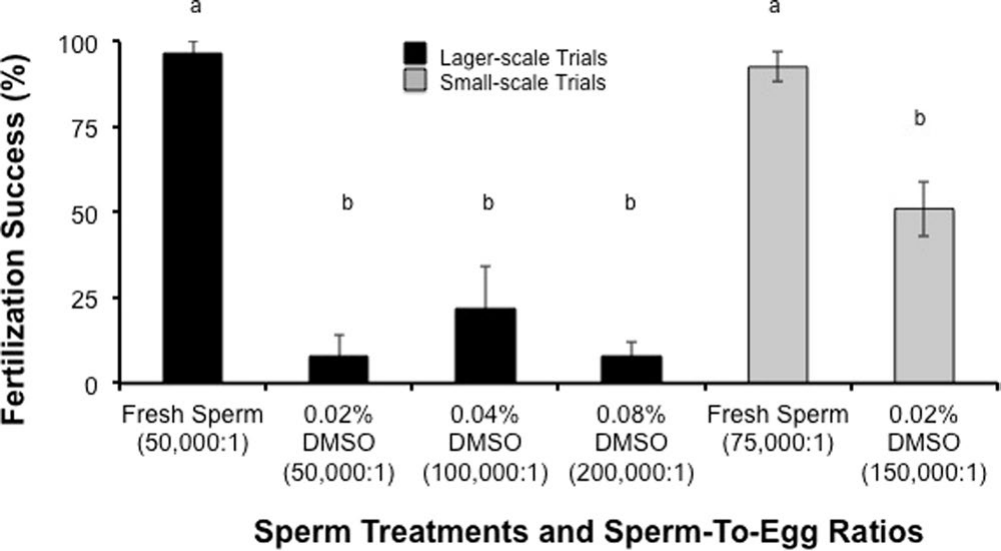
*In vitro* experiments comparing larger-scale and small-scale fertilization trials using *A. millepora*. Larger-scale fertilization trials are represented by the black bars. In these experiments, more cryopreserved sperm was added to each treatment while the egg number was kept constant. These examined the effects of increasing cryoprotectant concentration and sperm-to-egg ratio on fertilization success with the cryopreserved sperm (n = 2 individual egg donors and n = 4 to 6 pooled sperm donors, each treatment). The mean fertilization success with fresh sperm was >90% while using cryopreserved sperm the fertilization success was <25%. In comparison, *A. millepora* small-scale fertilizations trials (grey bars, n = 4 to 5 egg donors and n = 4 to 6 pooled sperm donors each treatment) demonstrated similar fresh fertilization success (>90%), but higher mean fertilization success with the cryopreserved sperm (>50%). Bars represented the mean (±SE) and the bars with different small letters are different (P < 0.05, ANOVA, F = 194). DMSO = dimethyl sulfoxide.

Scaling up the fertilization processes from small to larger scale processes to produce 1000’s of fertilized eggs proved to be relatively difficult, and we had many failures with no fertilization in the cryopreserved bowls. Success was only achieved when we paid attention to both the sperm concentration as well as the sperm-to-egg ratio. However, because of our cryopreservation methods, increasing sperm concentration led to parallel increases in cryoprotectant concentration. Specifically, Fig. 2 demonstrated that the larger-scale production achieved a mean fertilization success with cryopreserved sperm of ≤25% which was only half that observed in the small-scale trials. Maintaining the egg number relatively constant/treatment and using increasing sperm concentrations increased the sperm-to-egg ratio in the treatment, but also caused an increase in of dimethyl sulfoxide concentration (Fig. 2). A small-scale treatment with fewer eggs and a sperm-to-egg ratio of 150,000:1 and a cryoprotectant concentration of (0.02%) doubled the fertilization success to a mean of 50% (Fig. 2). Therefore, for cryopreserved sperm both the sperm-to-egg ratio and effective concentration of the dimethyl sulfoxide impacted fertilization success.

**Figure 3:**
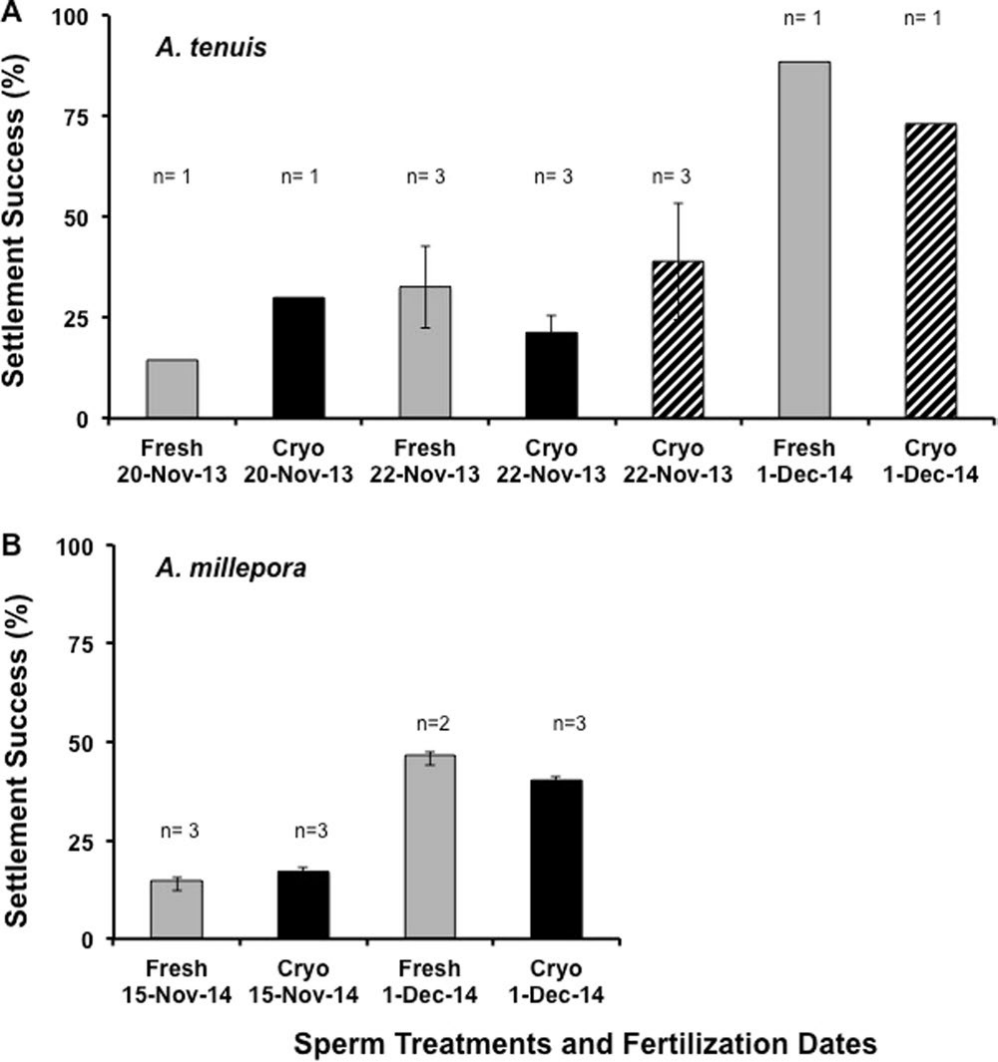
Mean (±SE) coral larval settlement success of *A. tenuis* (**A**) and *A. millepora* (**B**) produced in 2013 and 2014 from fresh (grey bars), freshly cryopreserved (2013 or 2014, black bars) or 1 to 2 year old cryopreserved sperm from 2012 (represented by the striped bars). The number of settlement plates in each treatment was indicated above each bar, however in each of the five fertilization trials several individuals were used (n = 2 to 5 egg donors and n = 4 to 6 pooled sperm donors). There was no difference between the treatment groups from the same dates, however, settlement success varied from month-to-month and year-to-year.

### Experiment #2: Settlement Success Is Not Impacted By Cryopreservation

**Figure 4:**
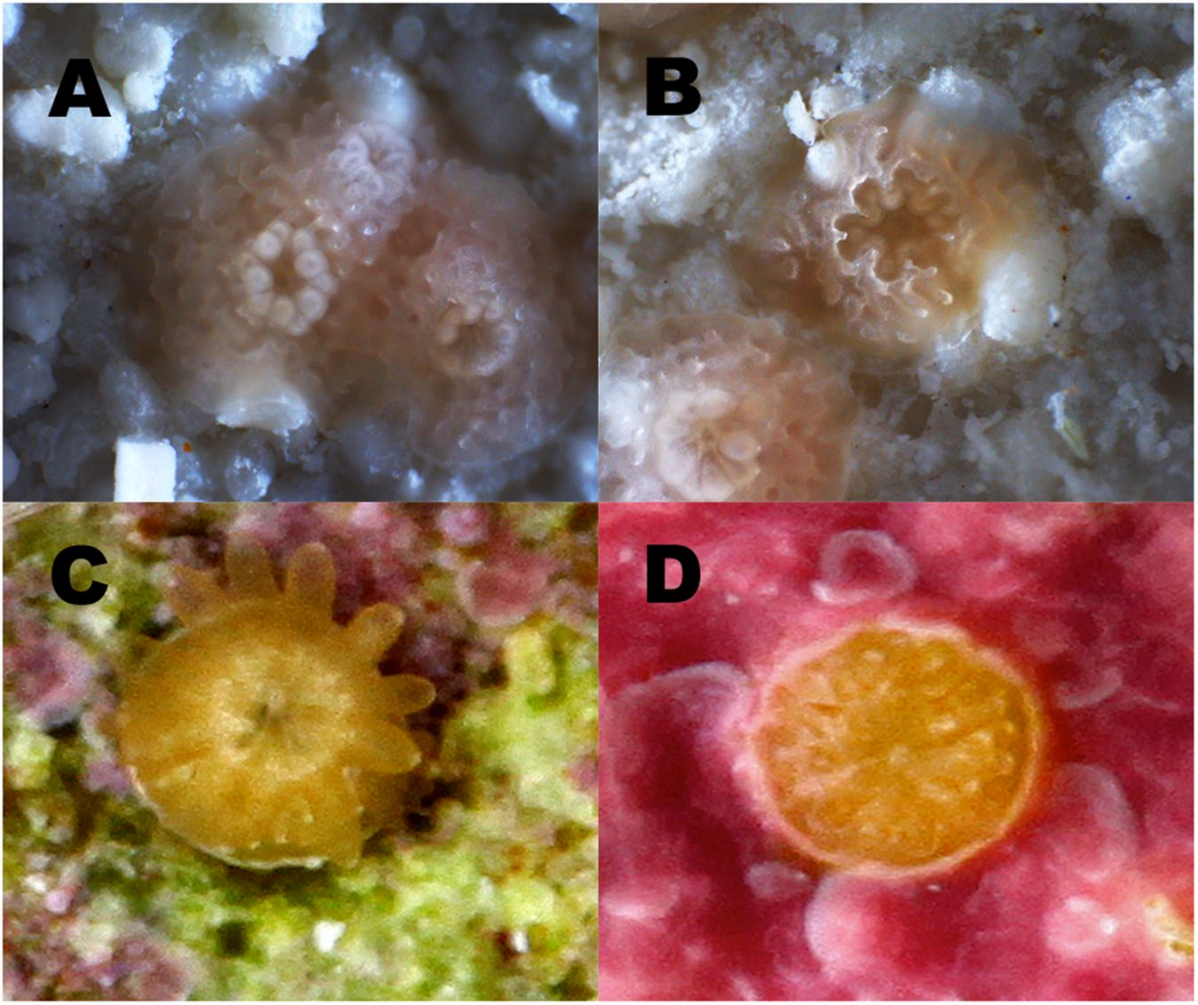
Coral settlers from *A. tenuis* (**A** and **B**) and *A. millepora* (**C** and **D**) demonstrated similar settlement size (~2 mm in diameter), complexity of their septa, tentacle development and calcareous skeleton, regardless of whether they were fertilized with fresh (**A**, **C**) or cryopreserved (**B**, **D**) sperm. The *A. millepora* images were taken at approximately 4 weeks post-fertilization and the settlers had acquired their *Symbiodinium*. The images of the *A. tenuis* settlers were taken at 2 weeks post-fertilization and had not yet acquired their symbionts. Bar = 1 mm.

Once the larvae had grown in the larval rearing chambers for ~5 days, they were placed into 50 L aquaria with flowing 0.04 µm filtered seawater (FSW). Settlement was highly variable within and between the species (Fig. 3). For example, the settlement success of *A. millepora* produced with fresh, unfrozen sperm was twice as high in Dec 2014 compared to Nov 2014 (Fig. 3B). This was a year with a split-spawn, so the quality of the gametes may have been inferior in the first month of the split spawn. However, there was no difference in the settlement success of the larvae produced with fresh and cryopreserved sperm within each month. This was based on two lines of examination of the data. During 2013, a single settlement experiment (*A. tenuis* with settlers produced from all three treatments: fresh pooled sperm, n = 3 settlement plates; cryopreserved pooled sperm less than 1 month old, n = 3 settlement plates; and cryopreserved pooled sperm that was 1 year old, n = 3 settlement plates) was analyzed, and there was no difference in their mean settlement success (p > 0.05, Kruskal-Wallis). Second, if all the experiments were pooled into two treatments (fresh sperm, n = 10 settlement plates; cryopreserved pooled sperm, n = 14 settlement plates) and normalized, their mean settlement success was similar; with fresh sperm producing 27.1 ± 0.1% and cryopreserved sperm producing 32.6 ± 0.3% settlement success (p > 0.05, unpaired t-test, F = 1.1). Taken together, these data suggest that cryopreservation does not negatively impact settlement. Specifically, the larvae from both species produced with fresh and cryopreserved sperm created their septa properly, laid down their calcareous skeleton similarly and absorbed *Symbiodinium* (Fig. 4). Moreover, the length of time within our biorepository did not matter, as the sperm that was frozen for less than 1 month produced similar settlement success as sperm that had been cryopreserved for up to 2 years.

## Discussion

Decisive conservation actions are needed to ensure persistence of coral reefs into the future, with the first priorities being reduction of CO_2_^14^ and habitat preservation. However, corals face pressing global and local threats, requiring future-thinking tools, including *ex situ* conservation practices. These include maintaining corals in live banks in zoos and aquaria and the formation of frozen biorepositories that can protect extant species as well as genetic diversity and integrity. In this context, we (and others) have argued for the biological banking of coral and their symbionts^5,15^. These resulting biorepositories may provide a major hedge against extinction for corals facing the damaging effects of climate change, disease and loss of genetic diversity.

An exciting development is the innovation of potential new genetic tools for wildlife is assisted evolution. Assisted evolution^8,9^ and assisted gene flow^10,11^ approaches can potentially increase coral climate resilience. The genetic adaptations of a population that result from a specific threat may be preserved in successive generations while re-introducing overarching gene diversity, leaving the population in a stronger position to face future threats, not related to the current wave^16^. However, if steps are taken to describe and bank population gene profiles when a threat is first identified, these genes can be introduced to the wider population over generations to maintain diversity in the long term.

Most importantly, frozen biorepositories should not be viewed as a last-ditch effort. They are most valuable when used not only as biosecure archives, but as living repositories; constantly added to, with cells withdrawn as required for research and restoration. Cryopreservation helps maintain species diversity and can directly benefit wildlife populations. An outstanding exemplar is the infusion of black-footed ferret frozen sperm from founding members 20 generations removed from the current population^17^. As reproduction becomes more impacted by increasing episodes of regional and global bleaching, the need for increased restoration processes will become critical^18^. Because the next 10 to 20 years may be perilous in terms of loss of coral species on our reefs, we need practical conservation solutions today that can preserve cells and their genes in excellent condition in even the most extreme conditions. Cryopreservation is one of the few technologies that can achieve this, and is ensuring the maintenance of reef biodiversity in a cost-effective manner for potentially hundreds of years.

Seed banks provide another great example of the value of biorepositories. Seed banks provide an invaluable role in helping to maintain our global food reserves that have helped us expand our ability to feed billions of people. Peres^19^ reviews the history of seed banks and the high-yielding agricultural varieties of the Green Revolution that led to ‘genetic erosion’ of food varieties. She advocates banking to preserve valuable genetic material, such as old landraces that will enable agricultural modernisation to proceed. With this model in mind, it is critical that we begin to bank coral genetic material that includes many local populations of coral species. If any type of genetic change to corals are to be achieved to help them become more resilient to climate change^8^, then a comprehensive repository with the widest genetic diversity for each species will be critical to this endeavour. Moreover, if the material in the biorepositories is going to be used (without modification) in the near future to help broaden existing diversity for shrinking populations, then methods to help create this high-throughput conservation action are key to these restoration efforts.

Small-scale fertilization trials have been used successfully on many species of coral to define the relative robustness of the cryopreserved sperm compared to the fresh sperm^6^. This paper identified the first settlement for coral larvae produced from cryopreserved sperm and steps to improve coral sperm cryopreservation to move us toward more comprehensive restoration methods using cryopreserved material. These steps include: 1) obtaining robust fresh sperm motility (ideally ≥ 80%) that can achieve post-thaw motility of cryopreserved sperm ≥ 40%; 2) using sperm-to-egg ratios ~100,000:1 to 150,000:1 for the cryopreserved sperm in the fertilization process; and 3) maintaining cryoprotectant concentration ≤0.02% during fertilization. Throughout most of the experiments in this paper, the starting fresh sperm motilities were >80%. However, with bleaching becoming more common in many oceans, these stressing events may have led to reduced sperm motility in many populations^20^, thus finding these robust populations with excellent fresh sperm motility challenging. Other factors, such as sperm-to-egg ratios were of critical importance for fertilization with cryopreserved sperm. However, increasing sperm-to-egg ratios increases cryoprotectant concentration during fertilization. Dimethyl sulfoxide has previously been identified as potentially interfering with fertilization^6^ and slowing development^20^. In this paper and others, the sperm was collected at 4 × 10^9^ cells/ml and diluted 1:1 with 20% dimethyl sulfoxide in FSW. In the future, this large first step dilution could be avoided by using a smaller volume of a higher concentration cryoprotectant, thus maintaining the sperm at a higher overall sperm concentration. When applied to the fertilization process, this protocol would then allow for a sperm-to-egg ratio of ~100,000:1 with a smaller added sperm and cryoprotectant volume, lowering the overall cryoprotectant concentration in the fertilization trial.

Although cryopreservation impacted coral motility and reduced fertilization success^6^, it did not alter the larval settlement success, as settlement was similar between groups produced with fresh and cryopreserved sperm on the same date. Similar settlement success between the larvae produced with fresh and cryopreserved sperm indicated that the damaging effects of the cryopreservation may have been repaired or the severely effected larvae died during the 5-day rearing period. Cryopreservation damage to cells and tissues is well known and can occur at each step of the cryopreservation process, including cryoprotectant exposure, dehydration, cooling and rehydration^21^. It can also cause damage to DNA^22^, and this damage has been specifically linked to the concentration of the cryoprotectant in some cells^23^. Thus, one of the most important aspects of improvement for coral sperm cryopreservation would be to produce a robust cryoprotectant that had less toxic effects than the dimethyl sulfoxide used here. Recently, coral sperm was cryopreserved using a solution containing sugars and methanol^24^ and may be promising in future trials.

Over the past 10 years, a global network of scientists, called the Reef Recovery Initiative, has broken new ground with scientific advances to conserve coral reefs using modern reproductive technologies, specifically cryopreservation. This novel approach stores coral tissue, cells and germ cells at very low temperatures to maintain their viability over hundreds of years. This effort has provided tools that can significantly contribute to the maintenance of genetic diversity of remaining coral populations. Spermatozoa from 16 different coral species have been successfully cryopreserved using the same standardized cryopreservation protocol^6^, including Hawaii coral (*Fungia scutaria, Montipora capitata*), Caribbean Coral (*Acropora palmata, Acropora cervicornis and Orbicella faveolata)* and Great Barrier Reef coral (*Acropora millepora*, *Acropora tenuis*, *Acropora loripes*, *Acropora hyacinthus*, *Platygyra lamolina*, *Platygyra daedalea, Acropora cytherea, Acropora florida, Acropora sarmentosa, Acropora nobilis* and *Goniastrea aspera*). Nevertheless, a great deal needs to be accomplished in order to make these biorepositories working-partners in active restoration on coral reefs.

The material in a repository can be held for tens of years, possibly hundreds of years. A good quality bank has a great many uses. It is the best quality source of genetic information for research, because the cells are alive with relatively undamaged DNA. However, consistent collections from existing populations must be ongoing to ensure that adaptive changes are represented in the bank. The progeny resulting from the bank can be used intermittently to restore gene diversity in small populations or after a sudden population decline, and a percentage of samples within the bank can be maintained in perpetuity against a catastrophic event^25^. The proportions and timing of each of these uses should be agreed with appropriate stakeholder groups at the time of collection. Finally, there must be some assurance that when the ultimate goals of the biorepositories are realized, that agreements will be put in place with management authorities as to the use of the material in the bank.

We believe that as the stressors and damage to reefs increase globally that our frozen repositories of sperm  and* Symbiodium* ^5^ and (on the near horizon) coral fragments, coral eggs and larvae may provide extraordinary tools to augment our conservation practices.

## Methods

### Gamete collection

Gametes from individual *A. tenuis* and *A. millepora* colonies were collected over three spawning seasons in 2012 and 2013 (only *A. tenuis* gametes were used) and 2014 (both *A. tenuis* and *A. millepora* gametes were used) from the south east corner of Esk Island (18° 46.420′ South, 146° 31.372′ East) under permit to the Australian Institute of Marine Science (AIMS) from the Great Barrier Reef Marine Park Authority (2012, AIM’s General Permit #G09/30237.1; 2013 and 2014, AIM’s General Permit: G12/35236.1). Whole colonies were transported in running seawater to AIMS in Townsville, Australia within hours. Each colony was labeled and maintained in running 0.04 µm-filtered seawater (FSW) in 1000 L plastic tanks for the duration of the spawning event. A pump circulated water through the tubs to maintain high flow over the colonies. The same colonies were not re-sampled from year-to-year.

Each night, all colonies were examined for evidence of setting (when the egg-sperm bundle becomes visible in the opening of the polyp, presaging spawning). Any setting colony was transferred to its own separate bin in FSW. Approximately 5 ml of egg/sperm bundles from each colony were collected in a separate 50 ml plastic tube and the water reduced to 5 ml, yielding 10 ml total volume in the tube. The bundles separated with the buoyant eggs on the top and the concentrated sperm on the bottom of the tube. The sperm was removed from the bottom of the tube and transferred to a separate 50 ml plastic tube and maintained until assessment for sperm motility and concentration (see below for further details). Most samples were diluted 1:1 with 20% dimethyl sulfoxide to yield approximately 2 × 10^9^ cells/ml for future use. All solutions and dilutions were made with FSW, unless stated otherwise. No institutional ethical approval was required for any of the experimental research described herein. Nevertheless, all efforts were made to maintain the animals under optimal care and husbandry.

### Reproduction

Sperm motility, concentration and cryopreservation was assessed and accomplished according to the methods described in^6^. Once the sperm from an individual coral were removed, its eggs were placed into a cleaning sieve (plastic plumbing pipe ~5 cm wide × 8 cm high fitted with 100-µm mesh) and rinsed several times with FSW until the eggs moved freely within the sieve. After cleaning, the eggs from a single individual were placed in a 3 L bowl with FSW to keep them viable for downstream fertilization.

During each spawn, sperm was cryopreserved from each species for use in the *in vitro* studies. This sperm was held at liquid nitrogen temperatures (−196 °C) for various times periods from less than 1 night (*A. tenuis* and *A. millepora*), approximately 1 month (*A. tenuis* and *A. millepora*) or 1 or 2 years (only *A. tenuis*). The frozen *A. tenuis* sperm used in 2013 and 2014 was from a single identified batch of pooled sperm frozen in 2012 with post-thaw motility of 45% or greater. Originally, the frozen samples were transported by air and land in dry shippers in liquid nitrogen vapor from Townsville, Australia to the CryoDiversity Bank at Taronga’s Western Plains Zoo in Dubbo, a distance of 1775 km. Once there, the samples were transferred into tanks and held in liquid nitrogen and stored from 1 to 2 years. In 2013 and then again in 2014, sperm samples from the same night and freezing run of cryopreservation for *A. tenuis* in 2012 were transported back to AIMS in dry shippers for use in our *in vitro* fertilization trials.

### Experiment 1: Small and Larger-Scale *In vitro* Fertilization Success

**Figure 5:**
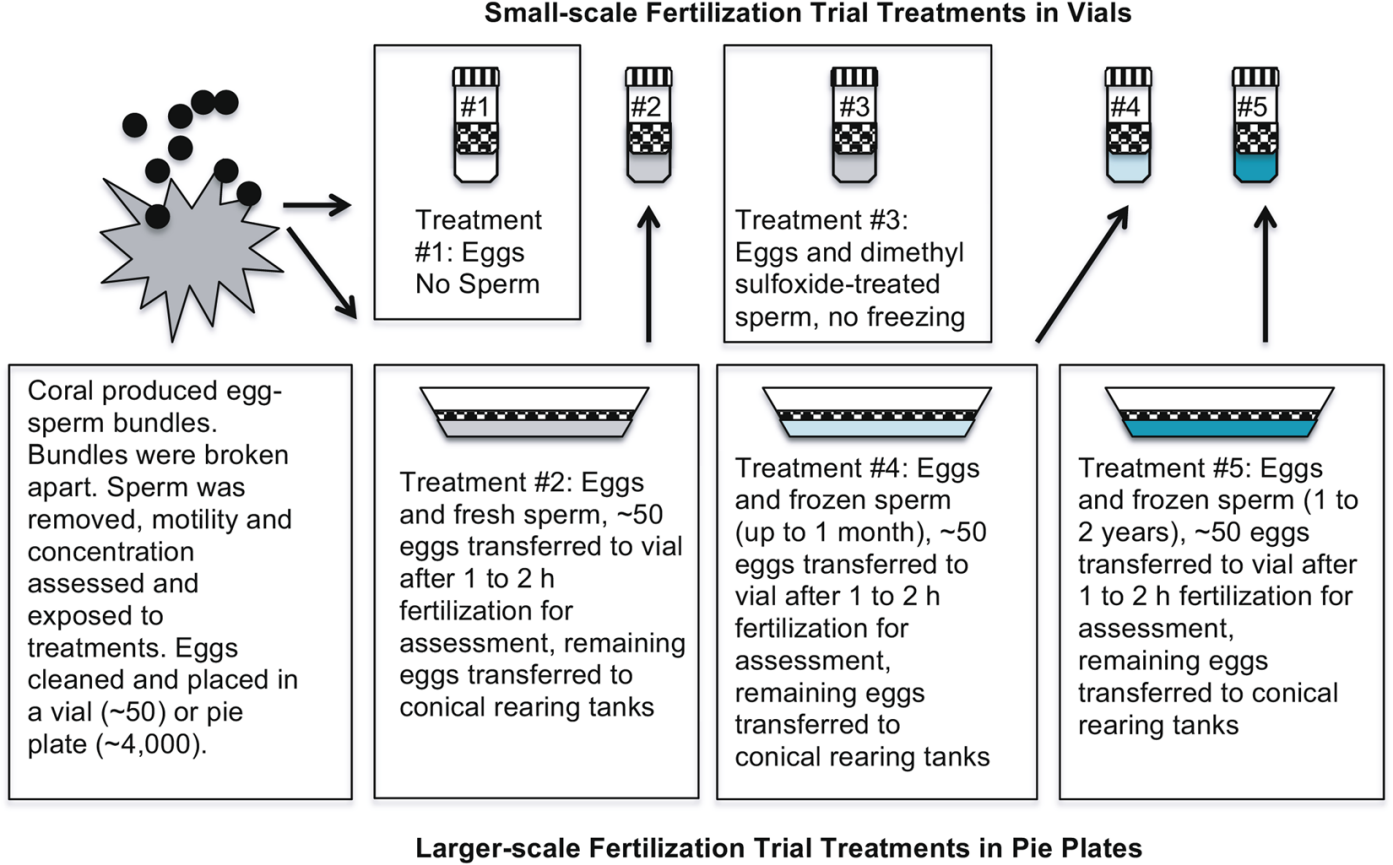
Flow diagram for small-scale and lager-scale *in vitro* reproduction in coral. Upper numbered vials represent the small-scale fertilizations trials that were done for various treatments, these included: #1) no sperm (white); #2) fresh sperm (gray) sampled from the lower larger-scale process; #3) fresh sperm (gray) mixed with the cryoprotectant to test toxicity to fertilization; #4) newly cryopreserved sperm (light blue, for ≤30 days or less) sampled from the lower larger-scale process; and, #5) older (~ for 1 to 2 yr) cryopreserved sperm (dark blue) sampled from the lower larger-scale process.

From our previous work^6^, we had defined small-scale fertilization methods for some acroporid species, but not for *A. tenuis* or *A. millepora*. These small-scale *in vitro* fertilizations were done in parallel with the larger-scale trials (Fig. 5). Briefly, *in vitro* fertilization assessments were conducted with 5 ml of FSW in a 20 ml glass scintillation vial, approximately 50 to 60 eggs and 5 µl of either FSW, fresh sperm (at 1 × 10^9^ cells/ml), or cryopreserved sperm (2 × 10^9^ cells/ml in the vial). The sperm concentration in the vial was diluted 1:1,000 to 1 or 2 × 10^6^ cells/ml, resulting in a sperm-to-egg concentration of ~1:100,000 to 1:200,000 in the vials and 0.01% dimethyl sulfoxide concentration in vials during fertilization. Treatments for the small-scale fertilization trials included: 1) eggs treated with no sperm added to assess self-fertilization; 2) eggs (sampled from the pie-plates, described below) treated with fresh sperm from that night (80 to 93% motility for both species); 3) eggs treated with unfrozen sperm from that night treated with 10% dimethyl sulfoxide to assess toxicity of the cryoprotectant; 4) eggs (sampled from the pie-plates, described below) with frozen sperm frozen up to 1 month and thawed; and, 5) eggs (sampled from the pie-plates, described below) treated with frozen sperm frozen from 1 to 2 yr and thawed (*A. tenuis* only).

Larger-scale fertilization trials were conducted to measure settlement success with *A. tenuis* in 2013 and with *A. tenuis* and *A. millepora* in 2014. In each species trial per night, the eggs from a single individual (n = 2 to 6 individual females/trial) were were divided into treatment groups (as in Fig. 5) and fertilized with fresh and cryopreserved pooled sperm (n = 2 to 6 pooled males) or no sperm.

To produce large numbers of fertilized eggs, an individual’s eggs were placed into either two or three glass pie-plates (23 cm diameter) with a total of 4,000 eggs/100 ml final volume. Fresh sperm (100 to 200 µl at 2 × 10^9^ cells/ml, yielding an sperm-to-egg ratio of 25,000: 1 to 50,000:1) or frozen and thawed cryopreserved sperm (200 to 800 µl at 2 × 10^9^ cells/ml, yielding a sperm-to-egg ratio range of 50,000: 1 to 200,000:1 and a dimethyl sulfoxide concentration of 0.02 to 0.8%) were thawed and quickly added to each bowl and the suspension gently stirred every 5 min over 1 h. Each treatment sequence was then repeated with eggs from 1 to 5 additional individual coral colonies each night, yielding 6 to 18 bowls during each experiment. Individuals within each sperm treatment (fresh or cryopreserved) were maintained separately during fertilization. After 1 to 2 h, the bowls were gently cleaned to remove excess sperm, ~50 eggs were sub-sampled from the pie-plates and transferred into 5 ml of FSW in the vials for later fertilization assessment (Fig. 5). The remaining eggs were transferred into the larval rearing system (described below). Approximately 4 to 8 h later, the percent fertilization of the within the vials was determined by counting the total number of eggs and embryos in each vial with embryos defined as those eggs that had gone through cleavage as observed using a dissecting microscope at 25 x magnification.

### Husbandry

All developing embryos and recruits were maintained in 2013 and 2014 in the National Sea Simulator at the Australian Institute of Marine Science in 0.04 µm FSW. After 2 h of fertilization, the developing coral embryos and unfertilized eggs (not all eggs successfully fertilized) from many individuals from a single coral species were placed into 60 L conical tanks with filtered seawater dripping in. Individuals from within each treatment (fresh or cryopreserved sperm) were combined in the larval rearing tanks, but all fresh and cryo-treatments were kept in separate rearing tanks- even those that spawned and were treated a few nights apart. The developing embryos were relatively fragile at this early point in development, so shear force from flow in the tanks was kept to a minimum. After 24 h the embryos were more robust, so aeration was started and water flow along the edge of the tank was increased to approximately 1 L/min causing the embryos to swirl in the water column. This helped to maintain the developing embryos and to remove degrading eggs from the tank. With this open-system rearing, a single 60-L tank could safely support up to 100,000 coral embryos and eggs, thus maintaining this density of larvae with comparable levels of survival (%) to a lower density system.

### Experiment #2: Comparing Settlement of Larvae Produced with Fresh and Cryopreserved Sperm

As part of this study, we wanted to begin to rear and produce settlers in larger numbers that might be used as the foundation for large-scale restoration efforts in the future. This necessitated using large settlement plates in large flow-through tanks. However, this limited the total number of replicates we could produce for each treatment. After 5 days, the developing coral larvae in the conical tanks were competent to settle^26^. Heavy-duty PVC plastic plates (40 by 22 cm) were drilled to flush-fit 165 aragonite plugs with stems (~2.5 cm on top) placed into a 50 L clear acrylic tank with gentle flow with 0.04 µm FSW (~1 L/min) and 100-µm mesh filtration to stop larval escape out of the tank. Two slightly different settlement processes were used. In 2013, crustose coralline algal chips (*Hydrolithon sp*.) were used to induce coral settlement within a few days following these methods^27^. In 2014, plugs had been preconditioned with *Titanoderma sp*. for over six months and were covered with the coralline algae. For each treatment from a particular fertilization date, an equal number of larvae (larvae produced with fresh or cryopreserved sperm) ranging from 780 to 7,700 were placed into the 50 L tank. The total number added into the settlement tanks depended on the fertilization success and the number of larvae at the end of the rearing treatment. After one week, the total number of recruits on each plug were counted, using a dissecting microscope (Wild M3, 10 x magnification). Settlement success was determined by the ratio of the number of total settlers divided by the total number of larvae placed into the tank.

### Data Analysis

All analyses in this study were performed using Graphpad Prism 6.0 (San Diego, CA). All percent data were scale arcsine transformed or normalized. Differences in the means were analyzed either with General Linear Models that were fit using either independent or dependent groups, depending upon the hypotheses being tested, sample sizes and observational periods. Dunnett’s post-comparison test was used for ANOVA analysis. For simplicity and future usefulness descriptive statistics are presented throughout as untransformed variables. A level of p < 0.05 was considered significant, and all data were expressed in mean (±SE).

The datasets generated during and/or analyzed during the current study are available from the corresponding author on reasonable request.
